# A Wearable Multidimensional Motion Sensor for AI-Enhanced VR Sports

**DOI:** 10.34133/research.0154

**Published:** 2023-05-25

**Authors:** Zi Hao Guo, ZiXuan Zhang, Kang An, Tianyiyi He, Zhongda Sun, Xiong Pu, Chengkuo Lee

**Affiliations:** ^1^ Beijing Institute of Nanoenergy and Nanosystems, Chinese Academy of Sciences, Beijing 101400, People’s Republic of China.; ^2^ School of Nanoscience and Technology, University of Chinese Academy of Sciences, Beijing 100049, People’s Republic of China.; ^3^ Department of Electrical and Computer Engineering, National University of Singapore, 4 Engineering Drive 3, Singapore 117576, Singapore.; ^4^ School of Mechanical and Materials Engineering, North China University of Technology, Beijing 100144, People’s Republic of China.

## Abstract

Regular exercise paves the way to a healthy life. However, conventional sports events are susceptible to weather conditions. Current motion sensors for home-based sports are mainly limited by operation power consumption, single-direction sensitivity, or inferior data analysis. Herein, by leveraging the 3-dimensional printing technique and triboelectric effect, a wearable self-powered multidimensional motion sensor has been developed to detect both the vertical and planar movement trajectory. By integrating with a belt, this sensor could be used to identify some low degree of freedom motions, e.g., waist or gait motion, with a high accuracy of 93.8%. Furthermore, when wearing the sensor at the ankle position, signals generated from shank motions that contain more abundant information could also be effectively collected. By means of a deep learning algorithm, the kicking direction and force could be precisely differentiated with an accuracy of 97.5%. Toward practical application, a virtual reality-enabled fitness game and a shooting game were successfully demonstrated. This work is believed to open up new insights for the development of future household sports or rehabilitation.

## Introduction

Innovating new technologies brings about a revolution in terms of life quality. In the past few decades, the rapid development of wearable electronics and the internet of things (IoTs) has brought us into the digital and intelligent health era [1–7], radiating to applications involving rehabilitation [8,9], health monitoring [10], disease diagnosis [11], etc. This also has a substantial impact on the sports field.

**Fig. 1.**
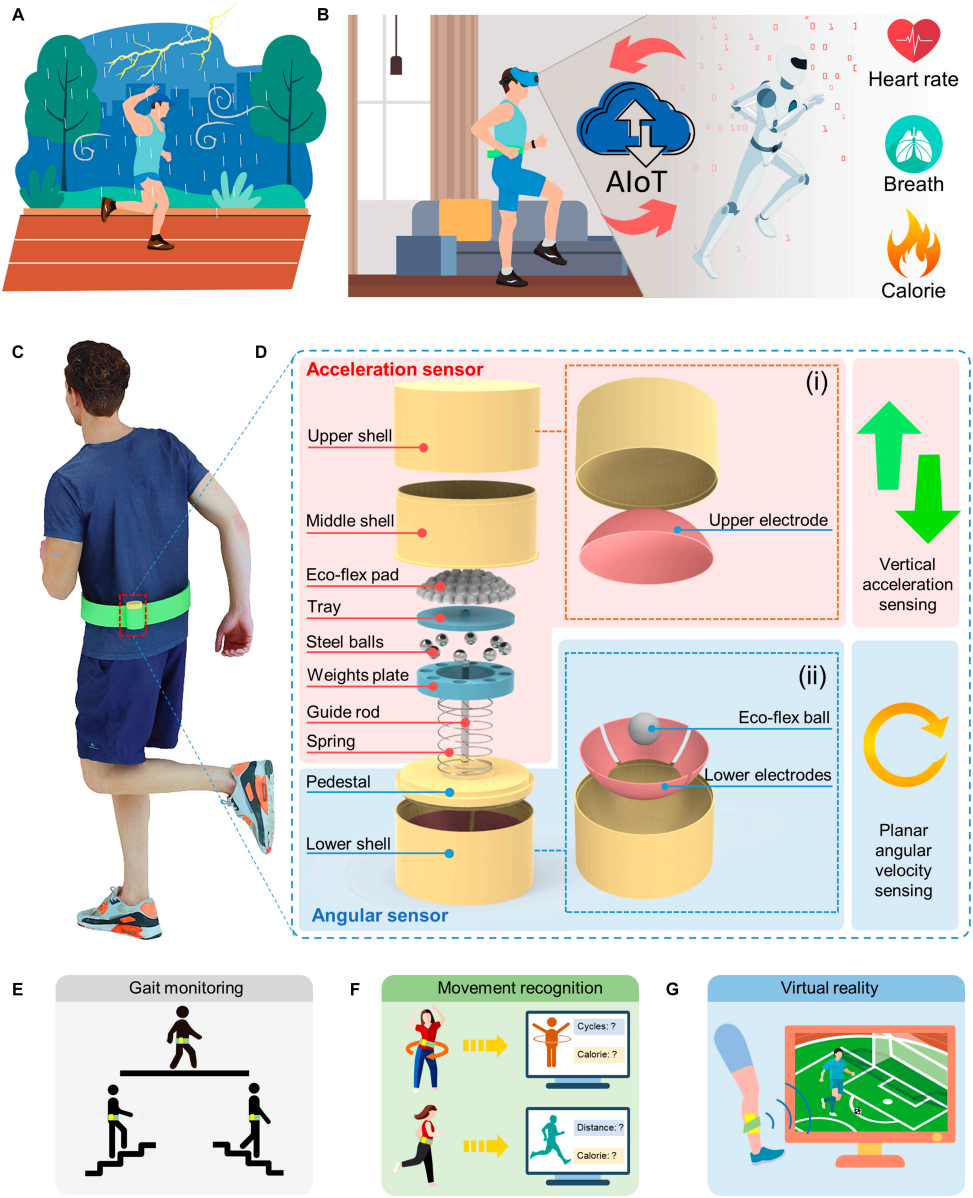
The schematics of the multidimensional sensor. (A) The influence of weather conditions on conventional sports events. (B) AIoT-enabled future household intelligence sports. (C and D) The structure of the multidimensional sensor. (E to G) Potential applications of the multidimensional sensor regarding gait monitoring, movement recognition, and virtual reality.

Regular exercise has been considered a key to open the door for a longer health span. It could delay the onset of 40 chronic conditions/diseases, such as cardiovascular disease, diabetes, or some mental diseases [12]. However, traditional sports events (e.g., running, hiking, football, etc.) are dominated by outdoor activities, which are significantly influenced by weather conditions (rainy, windy, temperature, etc.) or other special situations (e.g., coronavirus disease 2019 [COVID-19] pandemic) (Fig. 1A). In this case, doing exercises indoor or even working out at home is highly desired; this therefore promotes new commercial opportunities, typified by the Nintendo switch. They have developed a series of motion-sensing games through portable designs and achieved great commercial success, especially during the COVID-19 pandemic. One key component of these human–machine interface (HMI) systems should be the direction sensors that are integrated into the potable external devices (e.g., Joy-con) and could respond to angular velocity or accelerations. Unfortunately, relevant commercial angular velocity or acceleration sensors generally adopt piezoresistive or capacitive mechanisms, and power consumption issues seriously hinder their scalable utilizations [13]. In this regard, because of their miniaturized configurations, easy fabrication protocols, high output, and cost-effectiveness, the emerging triboelectric nanogenerators (TENGs) provide promising energy solutions [14–23]. Efforts have been devoted to developing self-powered TENG-based direction sensors for human health monitoring fields. For example, Xu et al. [24] have reported a spring-like TENG for vibration energy collection and acceleration sensing, Xie et al. [25] developed a self-powered angle sensor utilizing a rotation TENG, and Shi et al. [26] have constructed a self-powered gyroscope ball for both acceleration and angular velocity detection. However, the above-mentioned direction sensors mostly react to either vertical motion and acceleration, or planar motion and angular velocity. Limited detection dimensions as well as poor wearable comfort block their further applications. More importantly, existing strategies extract the motion information from obtained triboelectric signals by simply differentiating the signal amplitudes or peak numbers [27–29], which are not perfectly adapted to our unpredictable and complex daily movements. The burgeoning Artificial Intelligence of Things (AIoTs, the combination of artificial intelligence technology with IoT technology) provides a new strategy to unlock the shackles of technology [30–33]. Specifically, wearable electronic devices in IoT collect and transmit sensory data to the cloud, and the data are then remotely analyzed by artificial intelligence algorithms (e.g., machine learning or deep learning algorithms), which is an efficient way to learn higher-level features of the raw input signals, especially for the triboelectric featured dynamic sensing signals [34–36]. Besides, with the onset of virtual reality (VR) and augmented reality (AR), various gaming-based HMIs are developed [37–41]. Such functionality-enhanced HMI systems facilitate the user to absorb the virtual space that will pave a new way to construct the immersive VR sport system (Fig. 1B).

In this work, we proposed a self-powered wearable multidimensional motion sensor that can both sense vertical acceleration and planar angular velocity. Each part of the sensor (e.g., acceleration and angular velocity part) is sensitive to the mechanical stimuli and holds excellent durability (over 10,000 cycles). The multidimensional motion sensor was designed and developed to be integrated onto a belt for gait and waist motion sensing. By leveraging the Support Vector Machine (SVM) model and a classical machine learning algorithm for classification, the smart belt could identify various motion patterns including walking, running, waist spinning, and turning with an accuracy of 93.8%. Then, a VR fitness game was developed based on the smart belt. The virtual character was controlled by different movements and the calorie consumption of the controller during the exercise could be calculated instantaneously. Moreover, the multidimensional motion sensor could also be adopted to collect data with more abundant information. As evidence of the concept, we further wear the sensor at the ankle position to obtain the signals generated by shank motions that are more complicated than waist motions. Aided by the Convolutional Neural Network (CNN) model, kicking from different directions and forces could be effectively recognized with an accuracy of 97.5%. Hence, supported by the AI technique, the multidimensional motion sensor presented in this work could be used for gait monitoring, movement recognition, and VR, which not only shows its prospective application in the intelligence sports area but also exhibits great potential in the rehabilitation, smart home, and healthcare field (Fig. 1E to G).

## Results

### Design, sensing mechanism, and characterization of the multidimensional motion sensor

The configuration of the multidimensional motion sensor is schematically demonstrated in Fig. 1C and D. Generally, to detect motion along arbitrary directions, the multidimensional motion sensor is designed to equip with 2 parts: an acceleration sensor (Fig. 1D(i)) and an angular sensor (Fig. 1D(ii)). The acceleration sensor is assembled on the top of the whole structure, to detect vertical acceleration. It consists of an upper shell (the internal surface of which is attached with the upper electrode), a middle shell, an Eco-flex pad, a tray, steel balls, a weight plate, a guide rod, and a spring. As for the angular sensor, it is composed of a pedestal and a lower shell (inside of which contains 4 individual lower electrodes and an independent Eco-flex ball) for planar movement sensing or angular velocity sensing.

**Fig. 2.**
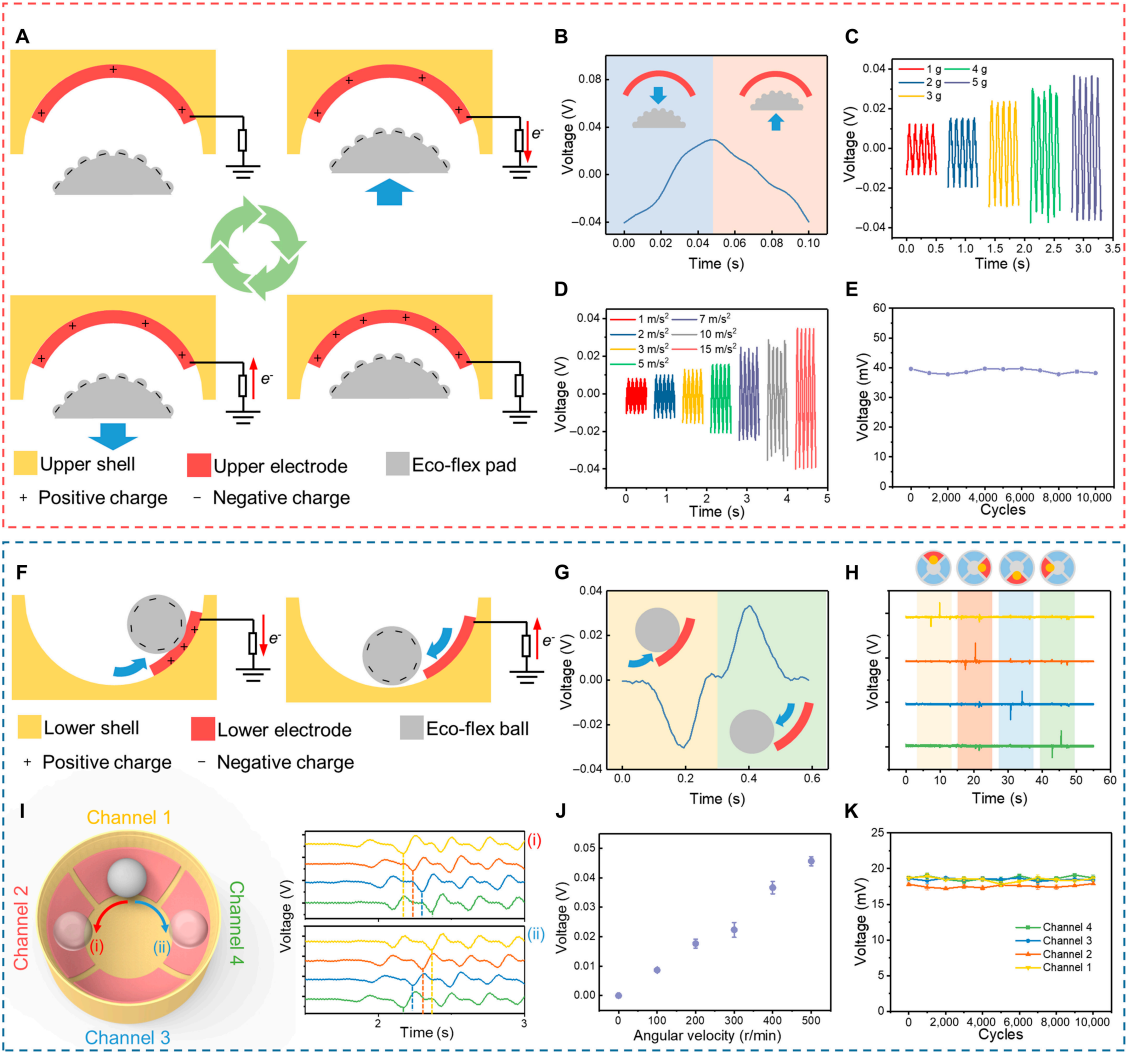
The mechanism and characterization of the multidimensional sensor. (A) The working principle of the acceleration sensor. (B) The typical voltage signal of the acceleration sensor. (C) Voltage outputs of the acceleration sensor when loaded with various weights. (D) The relationship between sensor output and acceleration value. (E) The durability of the acceleration sensor. (F) The working mechanism of the angular velocity sensor. (G) The typical voltage signal of the angular velocity sensor. (H) The independent output signals from 4 channels. (I) Outputs of the angular velocity sensor under spinning conditions. (J) The relationship between the angular velocity and sensor output. (K) The durability of the angular velocity sensor.

The working principle of the acceleration sensor is mainly based on the electrostatic induction effect, as shown in Fig. 2A. Due to the electronegativity, the Eco-flex naturally carried abundant negative charges. When the Eco-flex pad approaches (or even contacts with) the upper electrode under an external vertical acceleration, electrons will flow from the upper electrode to the ground. In contrast, when the Eco-flex moves away from the upper electrode, electrons will flow back to the upper electrode. To characterize the output performance of the acceleration sensor, an electrometer was adopted to measure the voltage output. The typically generated voltage signal is illustrated in Fig. 2B. The Eco-flex pad was fabricated through a mold casting method; the detailed process can be found in Fig. S1 and Materials and Methods. To increase the surface charge quantity of the Eco-flex pad, ball-shaped patterns were further created on its surface and the output could be increased to 125% of the initial state (Fig. S2). We also found that under the same acceleration, the increment of weight value (e.g., the number of steel balls) is beneficial to the sensitivity of acceleration detection (e.g., a higher output of sensor), as shown in Fig. 2C. Due to the limitation of the size of the weight plate, the maximum mass of steel balls could be loaded to 5 g. Then, the relationship between the acceleration value and sensor output was determined, and the results are demonstrated in Fig. 2D. As the applied acceleration increased, the outputs of the sensor also increased. A larger acceleration will create a smaller gap between the upper electrode and Eco-flex pad and therefore enhance the electrostatic induction effect. Besides, the durability of the acceleration sensor was also measured. After 10,000 cycles under the acceleration of 10 m/s^2^, the output of the sensor shows no obvious decrease (Fig. 2E).

The working principle of the angular sensor is similar to the acceleration sensor, as exhibited in Fig. 2F. When the Eco-flex ball rolls on the lower electrode, positive charges will be induced on the electrode, and electrons will flow from the electrode to the ground. When the Eco-flex ball leaves the electrode, electrons will flow back from the ground to the electrode to equilibrate the positive charges. Since the angular sensor contains 4 individual channels, we further adopted a multi-channel oscilloscope to simultaneously measure its voltage signals from each channel. The output waveform of the angular sensor from one single channel is demonstrated in Fig. 2G. The contact between the Eco-flex ball and the electrode will generate a negative peak and the Eco-flex ball generates a positive peak as it leaves the electrode. Besides, as shown in Fig. 2H, when the Eco-flex ball makes contact with each lower electrode from the original position (e.g., bottom of lower shell), respectively, every channel could work independently, and no obvious crosstalk was observed. Then, we further measured the output performance of the angular sensor under the spinning situation, as illustrated in Fig. 2I. If the angular sensor is spinning in an anticlockwise direction, the voltage waveform of channel 1 will be one-, two-, and three-quarters faster in phase than channels 2, 3, and 4, respectively. In contrast, in a clockwise direction, the phase of channel 1 will be slower than the other channels. Moreover, the relationship between the angular velocity and sensor output was investigated, as shown in Fig. 2J. The linear voltage output increases as the angular velocity increases. Figure 2K depicts the output voltage of the angular sensor after 10,000 cycles of spinning, showing the robustness of the sensor for longterm applications.

### Development of artificial intelligence-enhanced wearable sport system

**Fig. 3.**
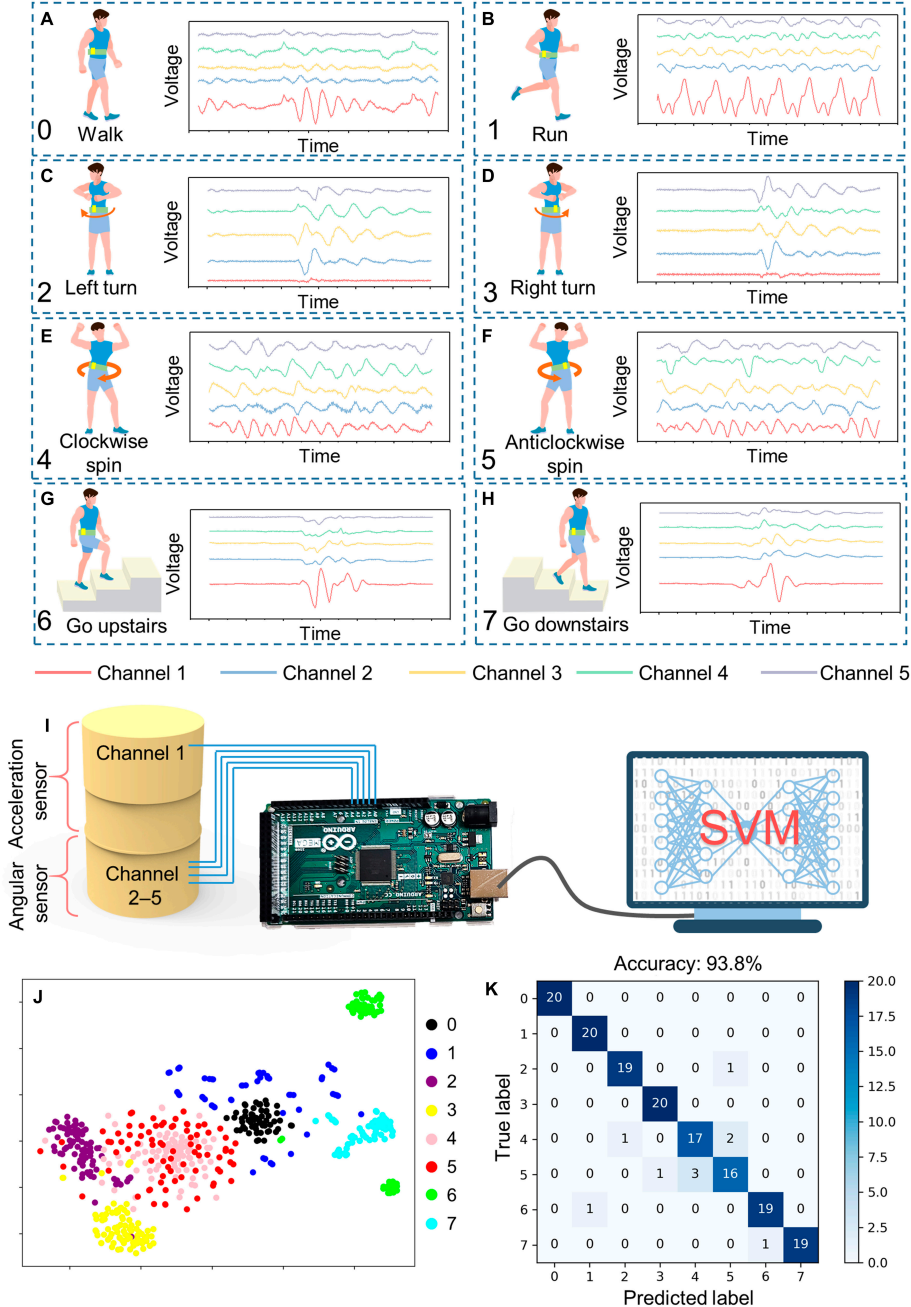
Recognition of waist motions. (A to H) Signals collected through Arduino that are generated by walking, running, left turning, right turning, clockwise spinning, anticlockwise spinning, going upstairs, and going downstairs. (I) The schematics of data collection and machine learning system. (J) The visualized clustered results of the dataset. (K) The confusion map of the classified result.

Considering the fact that wearable sensors that can be used to precisely gather motion signals from the human body during exercise and conventional sports events are susceptible to weather conditions, we built an indoor wearable intelligence sport system based on our multidimensional motion sensor. Since the waist is the joint that connects the upper and lower limbs, motions from different body parts will reflect on the waist motion [42,43]. In this regard, we integrated the multidimensional sensor with a belt and measured the signals at the waist position generated by the movement of walk, run, left turn, right turn, clockwise spin, anticlockwise spin, go upstairs, and go downstairs (marked from 0 to 7 in sequence), as shown in Fig. 3A to H. The data acquisition system is illustrated in a schematic diagram in Fig. 3I. For the instantaneous multi-channel data acquisition, each sensor channel would be connected to Arduino MEGA 2560 with 8 integrated circuits of amplifier. The channel of acceleration sensor was defined as channel 1, and the channels of angular sensor were set as channels 2 to 5. It could also be noticed that the collected triboelectric signals (Fig. 3A to H) have much information that was difficult to distinguish by the naked eye or simply differentiated by signal amplitude or peak numbers. Fortunately, as an emerging technique for extracting subtle features, machine learning has been commonly used in triboelectric signal analysis. The SVM is considered as a strong classification machine learning algorithm with the features of high efficiency, small sample input, and direct use [44–46]. Therefore, the raw data from the multidimensional sensor were further fed into the SVM model, which is constructed by Python for recognition tasks. A dataset containing 800 samples (each motion collected 100 samples) was constructed, where 640 samples were used for training (80%) and 160 samples were used for testing (20%). The data point length from each channel is set as 300; hence, there will be 5 channels × 300 = 1,500 features for each sample as the input of SVM. For the visualization, we utilized the t-distributed stochastic neighbor embedding (t-SNE) algorithm, a nonlinear dimensionality reduction technique for embedding high-dimensional data visualization in a low-dimensional space of 2 or 3 dimensions [47,48]. The clustered results of the dataset are successfully visualized as exhibited in Fig. 3J. After the training process, the new clustered results are demonstrated in Fig. S3, and it could be found that all data points from different motions are well classified. The recognition accuracy could reach up to 93.8%, and the confusion map of the classified result is shown in Fig. 3K.

However, for practical application, it is well known that triboelectric output will be susceptible to environmental variations (e.g., humidity, temperature) [49–51]. Also, we cannot ensure that the sensor will be worn at the exact same position as the last time used. All these issues will affect the signal waveform generated from the multidimensional sensor and decrease the recognition accuracy. To improve reliability, we attempted to extend a more comprehensive dataset from one single day to multiple days (Fig. S4), and the proportion of training and testing datasets is also kept at 80% and 20%. The dataset used in Fig. 3J and K was labeled as Day 0. When fed the dataset of Day 1 into the SVM model that was trained by Day 0, the accuracy decreased to 31.7% due to the different environmental conditions and positions (Fig. S4A). Nevertheless, as the number of days increases, the accuracy has significantly improved to 90% (Fig. S4B to F).

**Fig. 4.**
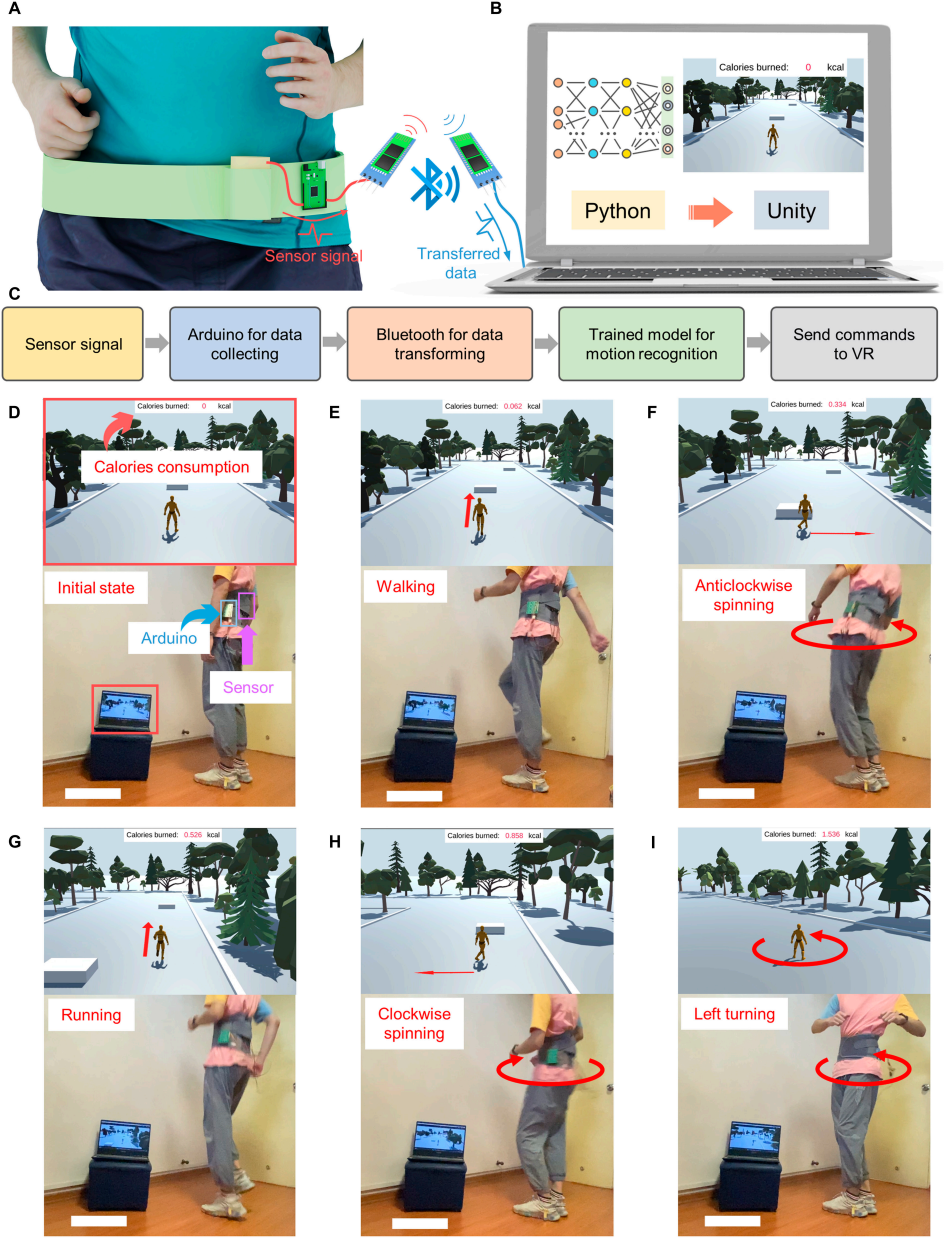
The VR fitness game based on the multidimensional sensor. (A and B) The schematic diagram of the control system. (C) The flowchart for the control process. (D) The photograph of the whole system and VR interface. (E to I) Demonstrations of virtual character controlling through gait and waist movement. Scale bars in (D) to (I) represent 35 cm.

Benefiting from the rapid development of VR/AR technologies, diversified immersive HMIs have been widely used in gaming, digital twin, social media, surgical training, etc. [52,53]. Here, we demonstrated a VR fitness game (constructed by Unity 3D) controlled by the multidimensional sensor integrated belt. The schematic diagram of the whole game system is exhibited in Fig. 4A to C. First, the triboelectric signal generated from waist motions will be gathered by Arduino. Then, the equivalent analog signal spectrum collected by Arduino will be transmitted to the PC through wireless transmission. Based on the received data, the SVM model that was trained by multiple days’ dataset could recognize the feature and send a command to Unity based on TCP/IP communication. This compels the virtual character to do corresponding actions. A photograph of the whole system and the VR interface is shown in Fig. 4D. In this fitness game, we choose 5 motions to control the virtual character. Motions of walking, anticlockwise spinning, running, clockwise spinning, and left turning were used for controlling the character to walk forward, go right, run forward, go right, and turn left, respectively (Fig. 4E to I). In the meantime, according to the data in Fig. S5, the calorie consumption during the exercise process was calculated and recorded simultaneously (Movie S1). In addition, this VR sport system also could have a rough distinction of waist-spinning velocity. For instance, we further measured the signals of fast/slow anticlockwise waist spinning and fast/slow clockwise waist spinning (labeled by 0 to 3, respectively), as shown in Fig. S6, and built a dataset following the same rules as before (e.g., each motion contains 100 samples, and the proportion of training and testing datasets is 4:1). After being trained by the SVM model, the recognition accuracy could reach 95% and the confusion matrix is depicted in Fig. S7. Based on the machine learning results, when we spin the waist at a low velocity, the character in VR space will have no reactions (Fig. S8A and C). In contrast, when spinning the waist at a high velocity, the character will move to the right or left according to the spinning directions (Fig.S8B and D). The whole process can be found in Movie S2.

**Fig. 5.**
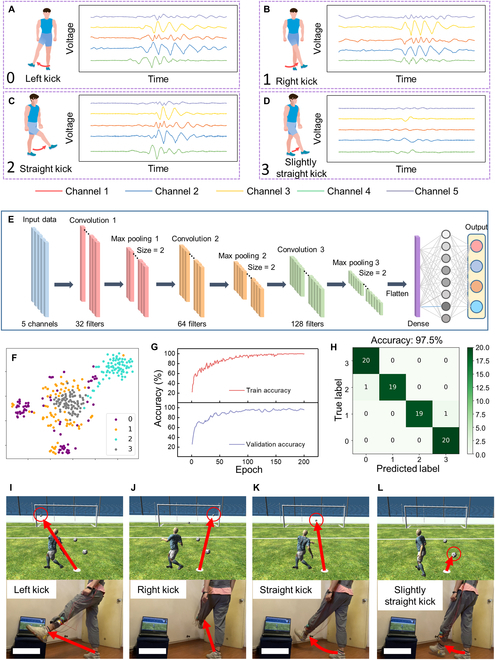
The application of the multidimensional sensor to detect shank movements. (A to D) Signals collect from motions of left kick, right kick, straight kick, and slightly straight kick. (E) The structure of the 1D CNN model. (F) The visualized clustered results of the dataset. (G) The accuracy variation of the training set and validation set, (H) The confusion map of classified results. (I and J) The demonstration of the VR football shooting game. Scale bars in (I) to (L) represent 35 cm.

Excellent motion sensors are expected to be available for arbitrary trajectories, not only for simple straight or circle trails but also for composite trails. The motions of the shank are the results of hip joint motions and knee joint motions, which should be more complicated than the waist motions [54,55]. Our multidimensional sensor could also be used to detect this complex movement. To prove it, we further wear the sensor at the ankle position to collect signals generated from shank motions. As demonstrated in Fig. 5A to D, signals of left kick, right kick, straight kick, and slightly straight kick (labeled by 0 to 3) are successfully obtained. Then, the collected data (each type contains 100 samples) were fed to the SVM model for machine learning (training set 80%, test set 20%), and the confusion matrix with a recognition accuracy of 80% is demonstrated in Fig. S9. The relevant low accuracy could be attributed to the abundant details of signals generated from shank motions and the limited computability of the SVM model.

Owing to the high level of performance across various types of data, deep learning technology has become a favored subset of machine learning, which could achieve a higher accuracy of complex recognition [56,57]. In particular, CNN technology of deep learning has been proven to be very effective for the overall dataset and suitable for analyzing time sequences of triboelectric sensor data [58,59]. In this regard, we constructed a one-dimensional (1D) CNN model for precisely recognizing shank motions. The detailed structure of the 1D CNN model is depicted in Fig. 5E, which includes 3 convolution layers, 3 max-pooling layers, and 1 fully connected layer that outputs the predicted result of 4 motions. Each type of dataset is separated into a training set (60%), a validation set (20%), and a testing set (20%). After 200 epochs, the accuracy of the training dataset could reach 100%, and the accuracy of the validation set could reach 98.3%, as shown in Fig. 5G. Then, the trained 1D CNN model could assist the testing set to achieve 97.5% accuracy of recognition, and the confusion map of classified results is exhibited in Fig. 5H. The t-SNE-aided visualization results of the dataset before and after training are shown in Fig. 5F and Fig. S10, respectively. Similar to the SVM model, to improve reliability, we further extend the dataset from 1 day to 5 days, and the accuracy could be increased from 77.5% to 95%, as illustrated in Fig. S11. As a practical demonstration based on the deep learning results, a football shooting VR game is designed and performed (Movie S3). In this VR scenario, the left, right, and straight kicks are set as the command for kicking the football to the left, right, and center areas of the goal (Fig. 5I to K). Besides, by determining the force of kicking, the distance of football trajectory can also be controlled. For instance, a slight kick will shoot the football to a closer distance (Fig. 5I).

## Discussion

In summary, a self-powered wearable multidimensional motion sensor is developed for intelligent indoor sports applications. Due to the integration of a vertical acceleration sensor and a planar angular velocity sensor, the multidimensional motion sensor could have a sensitive response to arbitrary movement trajectories. By combining with a belt, the multidimensional sensor can be used to detect waist or low limb motions, including waist spinning, turning, walking, and running. With the aid of the SVM machine learning algorithm, 93.8% accuracy in differentiating the above movements has been achieved. Furthermore, a VR fitness game controlled by the multidimensional motion sensor was demonstrated, and the calorie consumption during the whole exercise process was calculated and recorded. In addition, this multidimensional motion sensor is also competent to collect signals that contain more abundant information or details. Accordingly, the sensor was further worn at the ankle position to track the motion of the shank, and a high accuracy (97.5%) of identifying 4 motions including left, right, straight, and slight straight kicking was obtained through 1D CNN-based deep learning analytics. Looking forward, the multidimensional sensor offered an energy-saving and intelligent solution for movement trajectory tracking, which exhibits great potential in future intelligent home-based sports, VR gaming, and rehabilitation.

## Materials and Methods

### Fabrication of the multidimensional motion sensor

The upper shell, middle shell, tray, weight plate, pedestal, and lower shell were designed by 3D max and directly printed by a 3D printer (ANYCUBIC 3D-Printer 4Max Pro) using polylactic acid filament. Nickel conductive textiles were selected to be pasted on the inner surface of the upper and lower shells as upper and lower electrodes, respectively.

The Eco-flex pad and ball were fabricated by a mold-casting method. First, part A and part B of Eco-flex 0020 were mixed with a 1:1 weight ratio. Then, the obtained Eco-flex gel was poured into 3D-printed molds with patterned and ball shapes, respectively. After drying in an oven at 60 °C, the Eco-flex pad and ball were obtained.

### Measurement and characterization

The acceleration stimuli were provided by an exciter WA-0308. The angular velocity was controlled by a Spin Coater KW-4A. The output from the acceleration sensor was measured by a Keithley 6514 electrometer. Outputs of the angular velocity sensor were tested by an oscilloscope (DSO-X3034A, Agilent). Analog voltage signals generated from the multidimensional sensor were collected by Arduino MEGA 2560. The 1D CNN models were constructed in Python with Keras and TensorFlow backend.

## Data Availability

All data needed to evaluate the conclusions in the paper are present in the paper and/or the Supplementary Materials.

## Supplementary Materials

Supplementary 1 Fig. S1. The fabrication process of the Eco-flex pad. Fig. S2. The outputs of vertical mode sensors that adopted the patterned or original Eco-flex pad. Fig. S3. Cluster results of 8 movements’ datasets. Fig. S4. The accuracy of 6 movements (0: walking, 1: running, 2: left turn, 3: right turn, 4: clockwise spinning, 5: anticlockwise spinning) versus different days. Fig. S5. The calorie consumption of 4 movements. Fig. S6. The waveform of motions: (0) Fast anticlockwise spin, (1) Slow anticlockwise spin, (2) Fast clockwise spin, and (3) Slow clockwise spin. Fig. S7. The confusion matrix of SVM mode for (0) Fast anticlockwise spin, (1) Slow anticlockwise spin, (2) Fast clockwise spin, and (3) Slow clockwise spin. Fig. S8. The corresponding screenshot of slow anticlockwise spinning, fast anticlockwise spinning, slow clockwise spinning, and fast clockwise spinning in VR space of Unity. Fig. S9. The confusion matrix of SVM mode for (0) left kick, (1) right kick, (2) straight kick, and (3) slightly straight kick dataset. Fig. S10. Cluster results of 4 movements’ datasets (0: left kick, 1: right kick, 2: straight kick, 3: slightly straight kick) by SVM after training. Fig. S11. The accuracy of 4 movements (0: left kick, 1: right kick, 2: straight kick, 3: slightly straight kick) versus different days.
