# Supplementary material for: A Wearable Multidimensional Motion Sensor for AI-Enhanced VR Sports
Source: Research (Wash D C). 2023 May 25;6:0154. doi: 10.34133/research.0154 (PMC10211429; doi:10.34133/research.0154)
Supplement: Supplementary 1 — Fig. S1. The fabrication process of the Eco-flex pad. Fig. S2. The outputs of vertical mode sensors that adopted the patterned or original Eco-flex pad. Fig. S3. Cluster results of 8 movements’ datasets. Fig. S4. The accuracy of 6 movements (0: walking, 1: running, 2: left turn, 3: right turn, 4: clockwise spinning, 5: anticlockwise spinning) versus different days. Fig. S5. The calorie consumption of 4 movements. Fig. S6. The waveform of motions: (0) Fast anticlockwise spin, (1) Slow anticlockwise spin, (2) Fast clockwise spin, and (3) Slow clockwise spin. Fig. S7. The confusion matrix of SVM mode for (0) Fast anticlockwise spin, (1) Slow anticlockwise spin, (2) Fast clockwise spin, and (3) Slow clockwise spin. Fig. S8. The corresponding screenshot of slow anticlockwise spinning, fast anticlockwise spinning, slow clockwise spinning, and fast clockwise spinning in VR space of Unity. Fig. S9. The confusion matrix of SVM mode for (0) left kick, (1) right kick, (2) straight kick, and (3) slightly straight kick dataset. Fig. S10. Cluster results of 4 movements’ datasets (0: left kick, 1: right kick, 2: straight kick, 3: slightly straight kick) by SVM after training. Fig. S11. The accuracy of 4 movements (0: left kick, 1: right kick, 2: straight kick, 3: slightly straight kick) versus different days. [file research.0154.f1.docx]

**Front Matter**

Title

A wearable multi-dimension motion sensor for AI-enhanced VR sports

**Authors**

Zi Hao Guo^1,2,3^†, ZiXuan Zhang^3^†, Kang An^4^†, Tianyiyi He^3^, Zhongda Sun^3^, Xiong Pu^1,2,^*, and Chengkuo Lee^3,^*

**Affiliations**

^1.^ Beijing Institute of Nanoenergy and Nanosystems, Chinese Academy of Sciences, Beijing 101400, People’s Republic of China

^2.^ School of Nanoscience and Technology, University of Chinese Academy of Sciences, Beijing 100049, People’s Republic of China

^3.^ Department of Electrical and Computer Engineering, National University of Singapore, 4 Engineering Drive 3, Singapore 117576, Singapore

^4.^ School of Mechanical and Materials Engineering, North China University of Technology, Beijing 100144, China

*Corresponding authors: Chengkuo Lee, elelc@nus.edu.sg; Xiong Pu, puxiong@binn.cas.cn

† Zi Hao Guo, ZiXuan Zhang, Kang An have contributed equally to this work


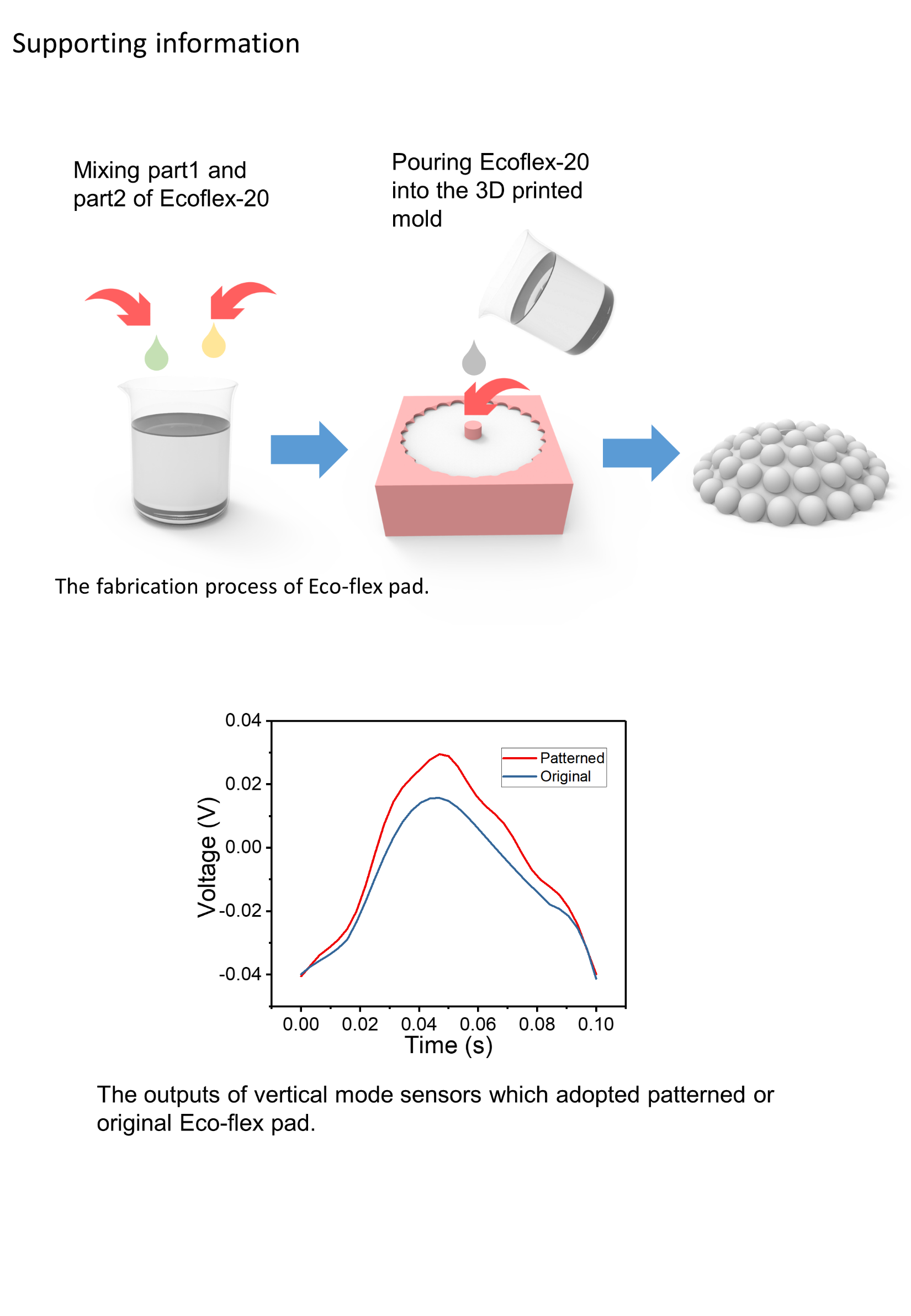


**Fig. S1.** The fabrication process of Eco-flex pad.


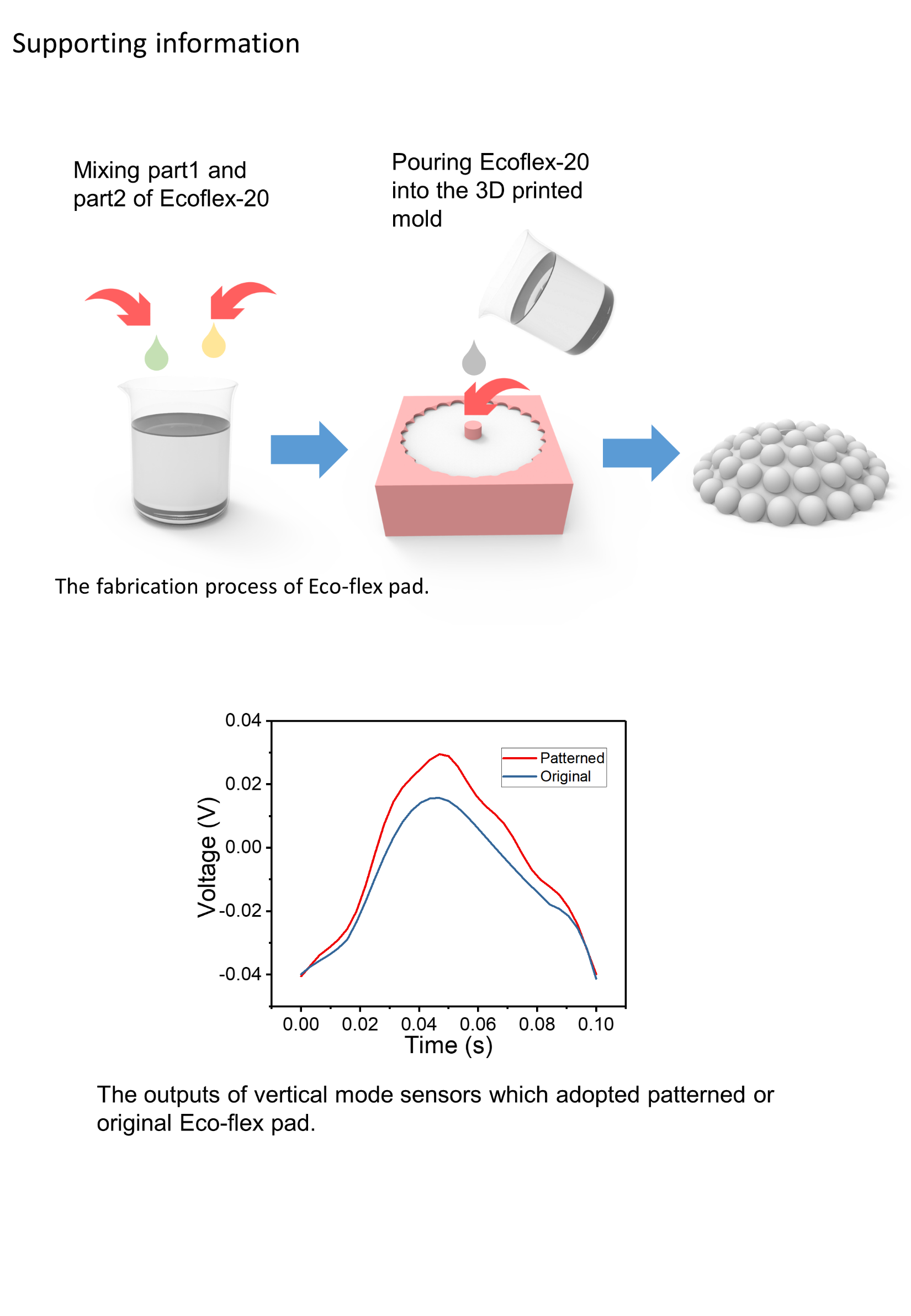


**Fig. S2.** The outputs of vertical mode sensors which adopted patterned or original Eco-flex pad.


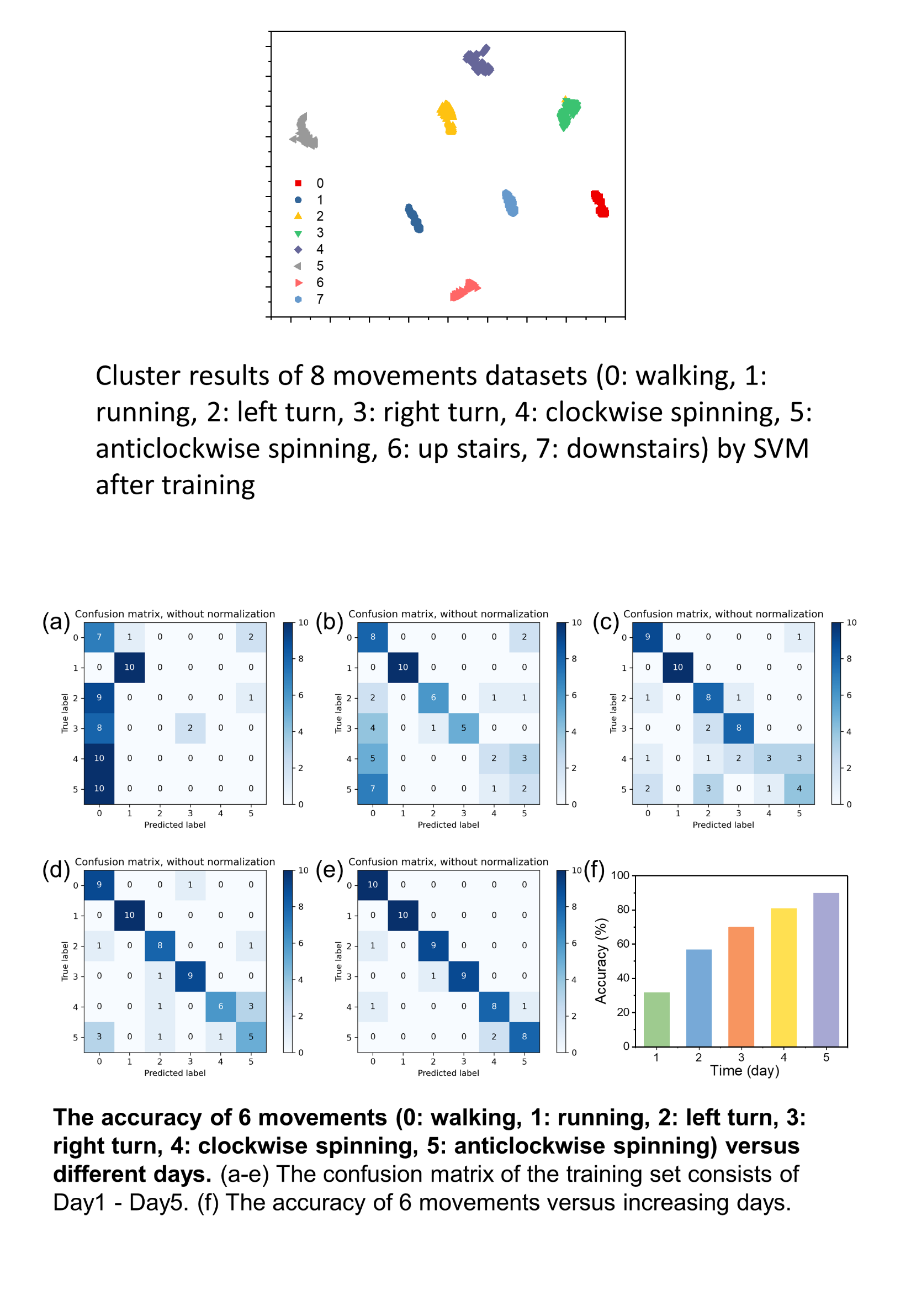


**Fig. S3.** Cluster results of 8 movements datasets (0: walking, 1: running, 2: left turn, 3: right turn, 4: clockwise spinning, 5: anticlockwise spinning, 6: upstairs, 7: downstairs) by SVM after training


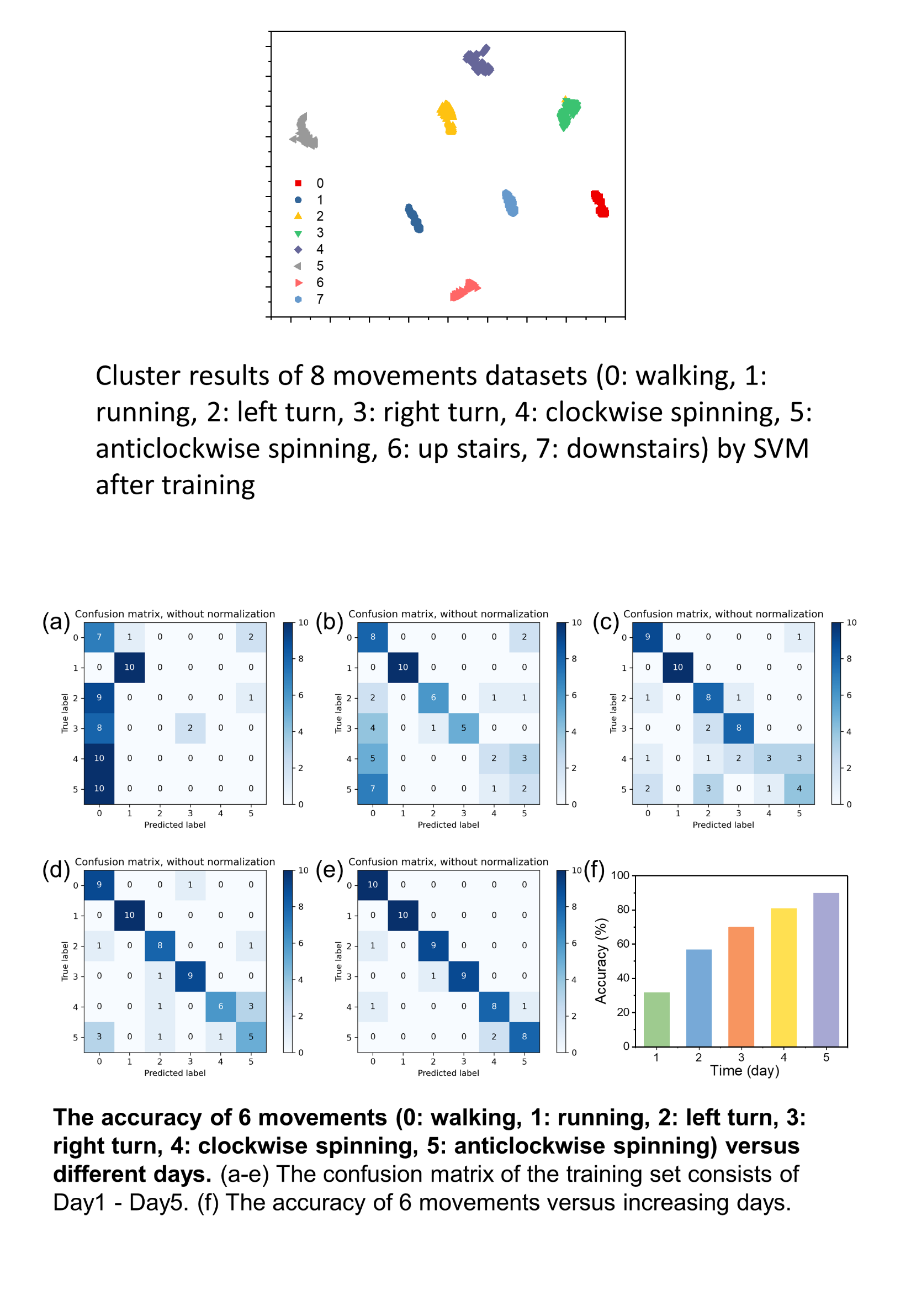


**Fig. S4.** The accuracy of 6 movements (0: walking, 1: running, 2: left turn, 3: right turn, 4: clockwise spinning, 5: anticlockwise spinning) versus different days. (a-e) The confusion matrix of the training set consists of Day1 - Day5. (f) The accuracy of 6 movements versus increasing days.


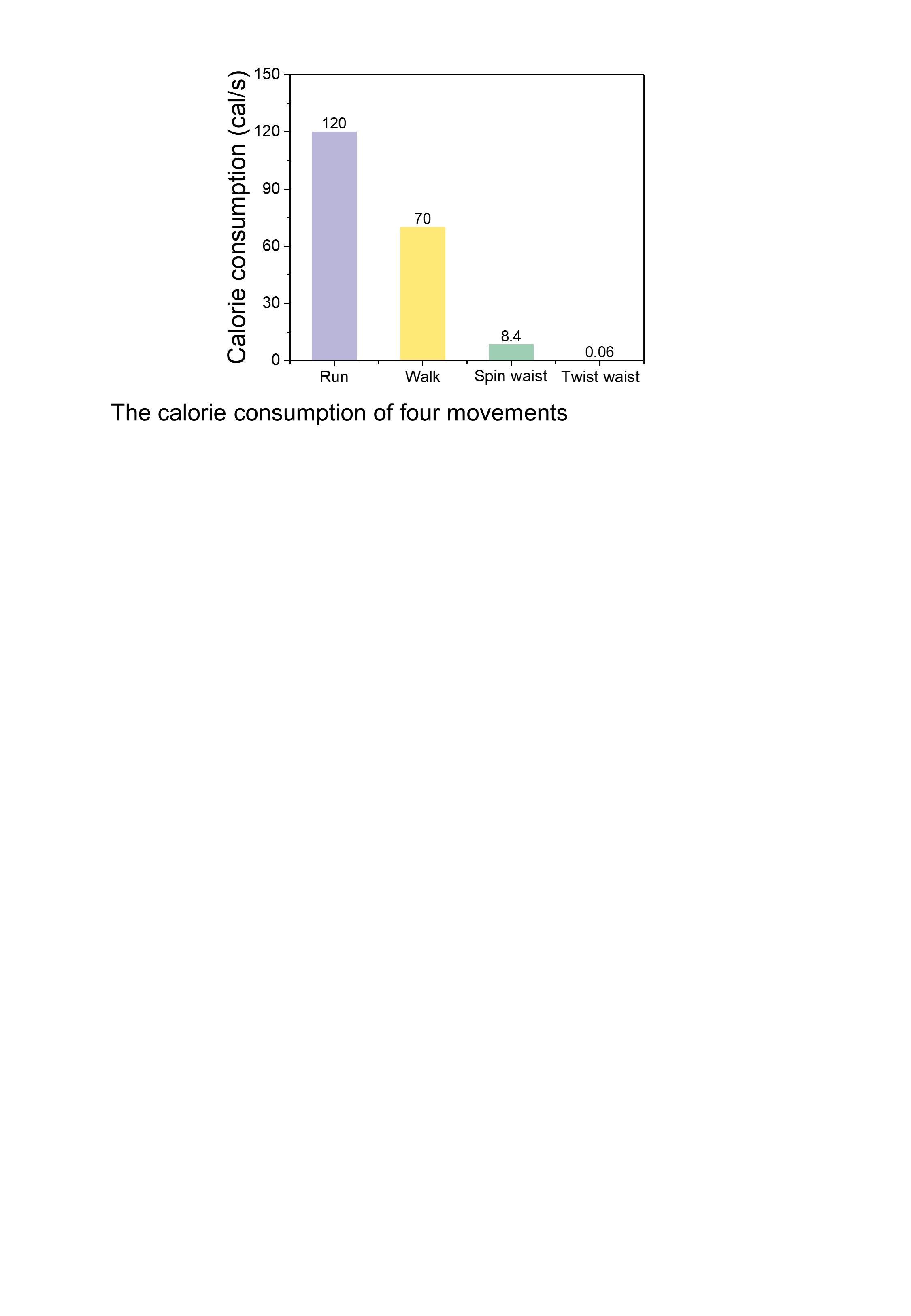


**Fig. S5.** The calorie consumption of four movements

References:

**Calories consumption of running and walking:**

Lester J, Hartung C, Pina L, et al. Validated caloric expenditure estimation using a single body-worn sensor[C]//Proceedings of the 11th international conference on Ubiquitous computing. 2009: 225-234.

**Calories consumption of waist spinning:**

Holthusen J. Relative exercise intensity and caloric expenditure of hooping[D]. , 2010.

**Calories consumption of waist twisting:**

https://livehealthy.chron.com/calories-burned-doing-torso-twist-9507.html


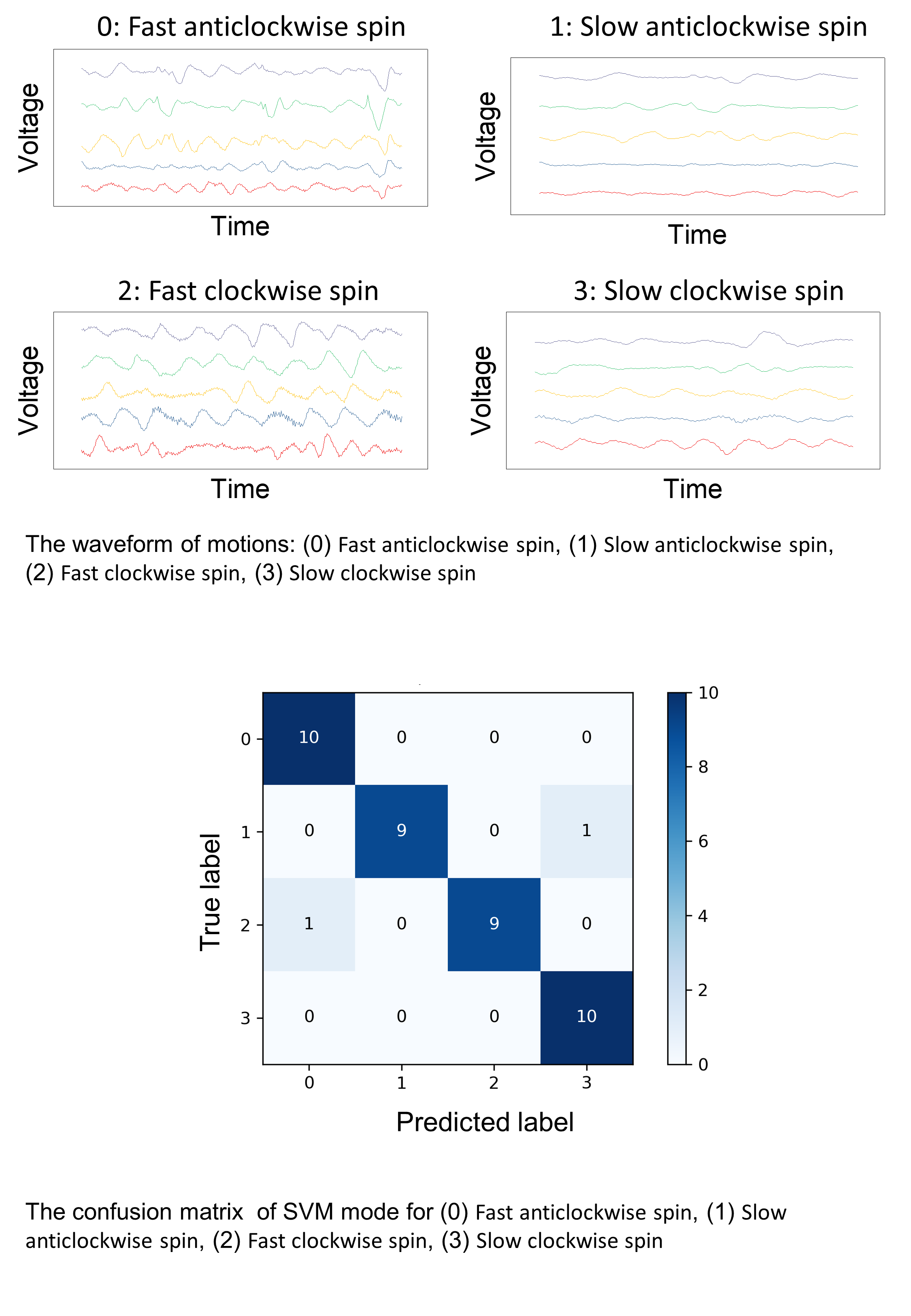


**Fig. S6.** The waveform of motions: (0) Fast anticlockwise spin, (1) Slow anticlockwise spin, (2) Fast clockwise spin, (3) Slow clockwise spin.


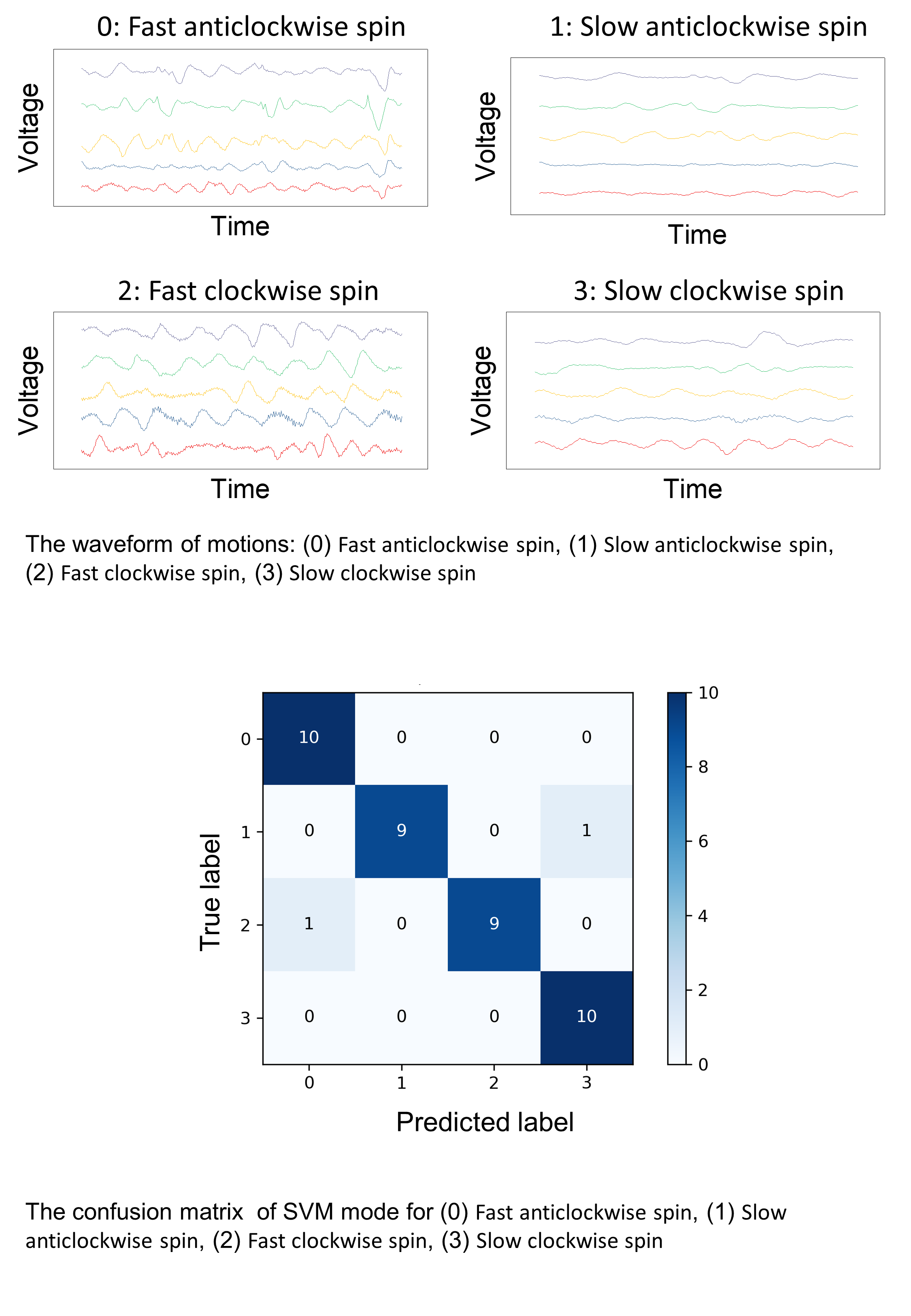


**Fig. S7**. The confusion matrix of SVM mode for (0) Fast anticlockwise spin, (1) Slow anticlockwise spin, (2) Fast clockwise spin, (3) Slow clockwise spin.


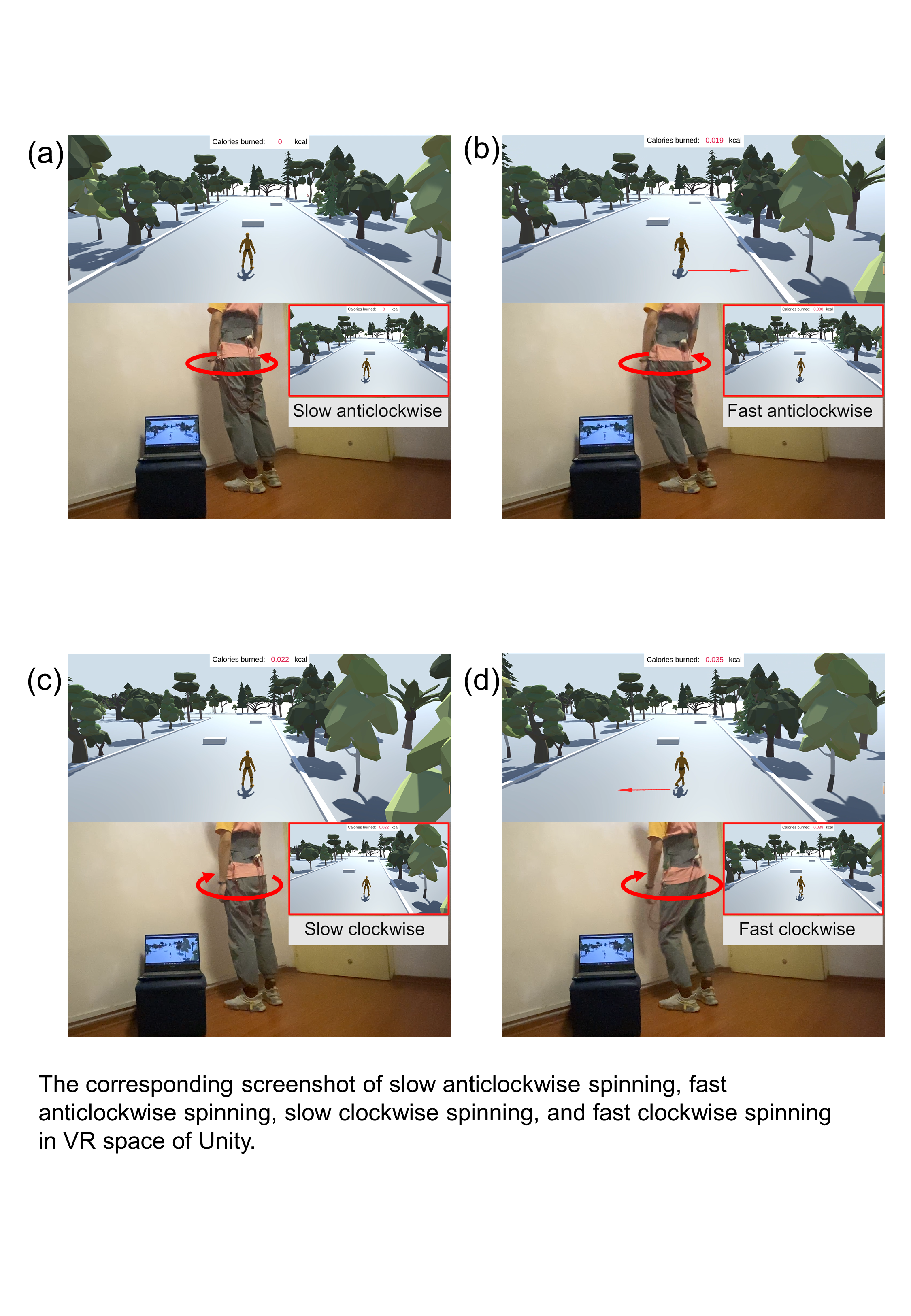


**Fig. S8.** The corresponding screenshot of slow anticlockwise spinning, fast anticlockwise spinning, slow clockwise spinning, and fast clockwise spinning in VR space of Unity.


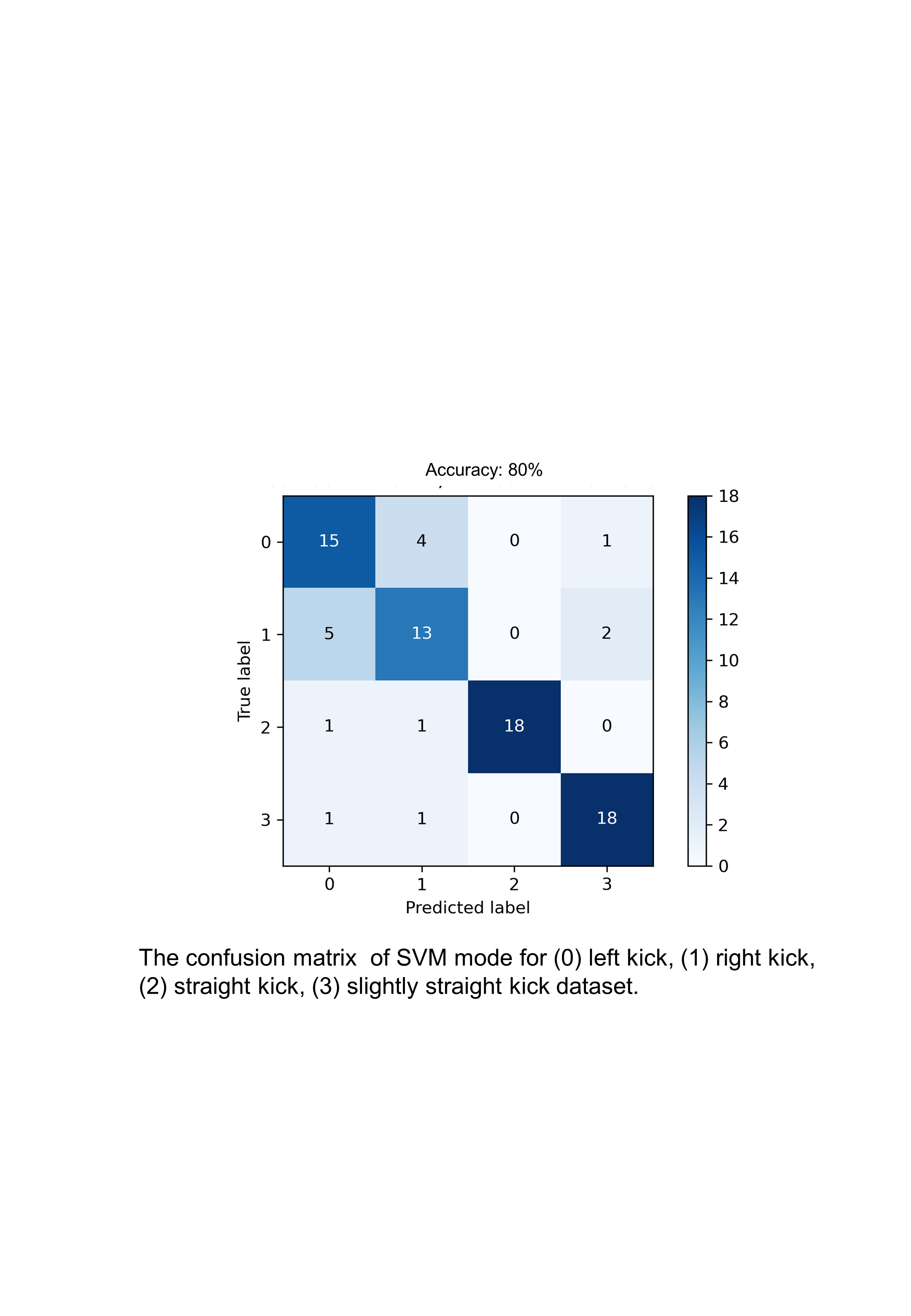


**Fig. S9.** The confusion matrix of SVM mode for (0) left kick, (1) right kick, (2) straight kick, (3) slightly straight kick dataset.


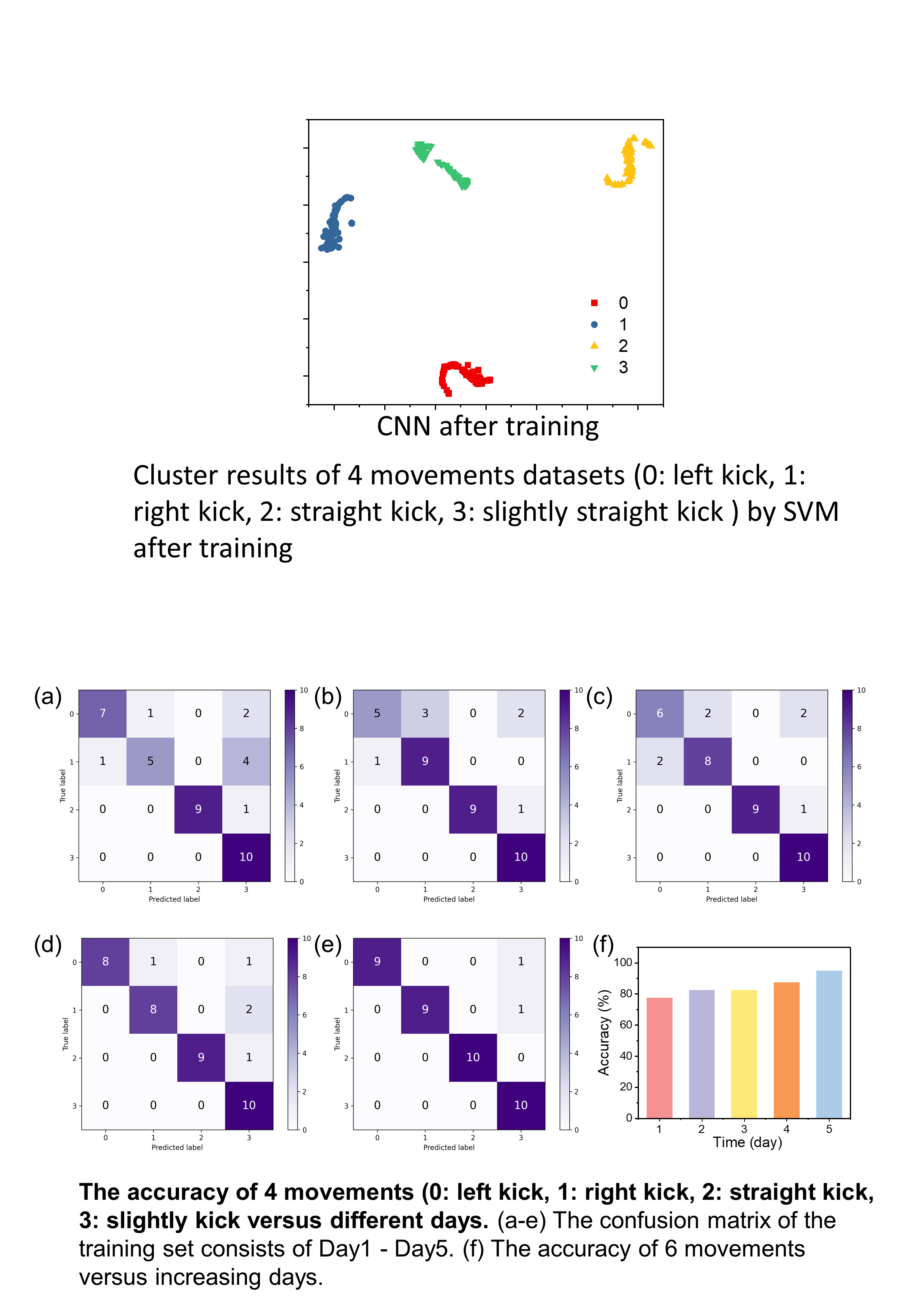


**Fig. S10.** Cluster results of 4 movements datasets (0: left kick, 1: right kick, 2: straight kick, 3: slightly straight kick ) by SVM after training.


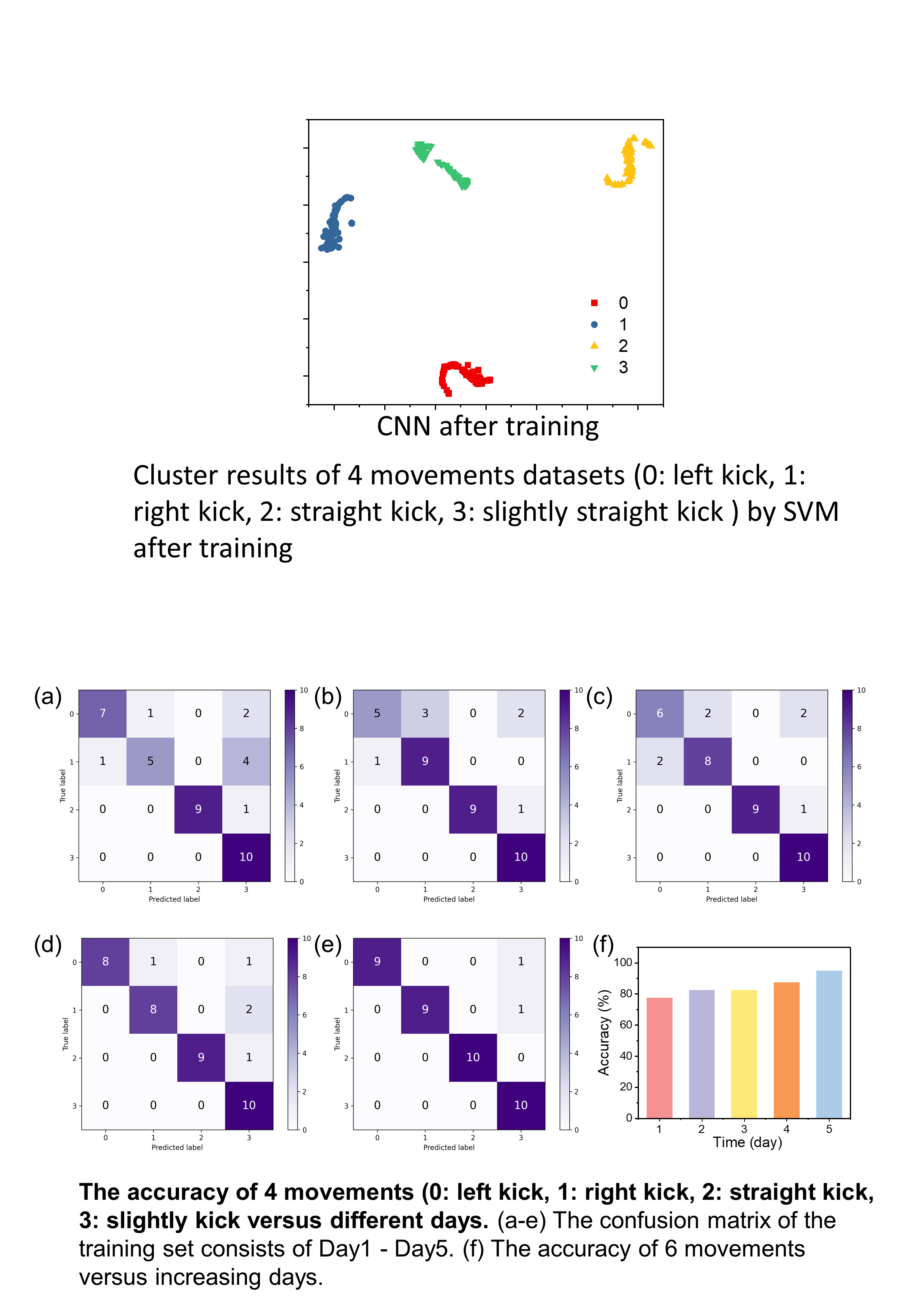


**Fig. S11.** The accuracy of 4 movements (0: left kick, 1: right kick, 2: straight kick, 3: slightly kick versus different days. (a-e) The confusion matrix of the training set consists of Day1 - Day5. (f) The accuracy of 6 movements versus increasing days.
